# GPX1 confers resistance to metabolic stress in BCR/ABL-T315I mutant chronic myeloid leukemia cells

**DOI:** 10.1038/s41420-025-02502-z

**Published:** 2025-05-09

**Authors:** Jun-Dan Wang, Jin-Xing Wang, Zhi-Li Lin, Na Xu, Ling Zhang, Jia-Jun Liu, Rui Gao, Zi-Jie Long

**Affiliations:** 1 Department of Hematology, The Third Affiliated Hospital, Sun Yat-sen University; Institute of Hematology, Sun Yat-sen University, Guangzhou, China; 2 Department of Pathology Technique, Guangdong Medical University, Dongguan, China; 3 Department of Hematology, Nanfang Hospital, Southern Medical University, Guangzhou, China; 4 Department of Oncology, The Third Affiliated Hospital, Sun Yat-sen University, Guangzhou, China

**Keywords:** Chronic myeloid leukaemia, Cancer metabolism

## Abstract

Chronic myeloid leukemia (CML) harboring BCR/ABL-T315I mutation has been a challenging obstacle for targeted therapy due to the acquired resistance to tyrosine kinase inhibitor (TKI)-based therapy. Thus, it is especially urgent to investigate more effective therapeutic targets to overcome T315I-induced resistance. Here, we reported that BCR/ABL-T315I mutant CML cells possessed a long-term proliferative capacity and tolerance to metabolic stress. Importantly, we also found that selenoamino acid metabolism was increased in the bone marrows of BCR/ABL-T315I patients compared with non-T315I patients by GSEA from RNA-Seq data. Indeed, GPX1 was highly expressed in T315I mutant cells, while knockout of GPX1 significantly suppressed cell proliferation and triggered apoptosis under glucose-deprived condition. GPX1 knockout showed decreased cell metabolism signaling as well as mitochondrial gene expression by RNA-Seq. Mechanistically, GPX1 maintained mitochondrial activity and oxygen consumption rate (OCR), retaining mitochondrial redox homeostasis and oxidative phosphorylation (OXPHOS). Additionally, mercaptosuccinic acid (MSA), a GPX inhibitor, inhibited CML colony formation and induced cell apoptosis under glucose-free condition. Therefore, GPX1 is a promising therapeutic target to overcome drug resistance induced by the T315I mutation, which provides a novel approach for BCR/ABL-T315I CML treatment by disturbing mitochondrial OXPHOS.

## Introduction

Chronic myeloid leukemia (CML) is a stem cell-derived hematological malignancy with genomic aberrations appearing in hematopoietic stem and progenitor cells characterized by a fusion oncogene BCR/ABL. Due to the success of tyrosine kinase inhibitors (TKIs) such as imatinib, nilotinib and dasatinib, the satisfactory therapeutic effects has been achieved. However, the presence of BCR/ABL gene mutations often results in drug resistance and relapse [1]. The T315I mutation is the most common type, leading to resistance to first and second-generation TKIs. Although ponatinib, a third-generation TKI, can overcome the resistance caused by the T315I mutation, the severe cardiovascular toxicity profile limits its clinical application [2]. Yet, major challenges still stand in the way of improving T315I-CML patient outcomes and quality of life. Therefore, the development of more optimal therapeutics for treating T315I-CML patients is urgently needed.

CML leukemia stem cells (LSCs) can be considered as drug-resistant cells, which is BCR/ABL kinase-independent [3]. Thus, the study of BCR/ABL-independent features unique to mutant leukemia cells would be a particularly potential strategy for CML therapy. Nowadays, cellular metabolic reprogramming is considered to be associated with leukemogenesis and drug resistance in myeloid leukemia, especially oxidative phosphorylation (OXPHOS) [4]. Most anabolic and catabolic pathways, including the biosynthesis of amino acids, nucleic acids, lipids, and cofactors like NADH and NADPH, occur within mitochondria [5]. Leukemia cells often undergo mitochondrial metabolic reprogramming to adapt to rapid proliferation or enhance tolerance to environmental challenge [6]. Previous study reports that compared to the CD34^-^ CML LSCs, upregulated TCA cycle flux and oxidative metabolism are observed in CD34^+^ counterparts [7]. The diverse function of mitochondria enables tumor cells to adapt to the dynamic microenvironment and provide them with a high degree of flexibility to promote cell growth and survival [5]. By a single-cell RNA sequence analysis, OXPHOS related genes are enriched in BCR/ABL^+^ LSCs compared to hematopoietic stem cells [8]. Thus, LSCs or drug-resistant cells, appear to be OXPHOS-dependent to acquire malignant capacity.

Glutathione peroxidase 1 (GPX1), one of the most abundantly expressed members in the GPX family, primarily participates in hydrogen peroxide conversion, metabolism and redox homeostasis [9]. GPX1 is also an antioxidant enzyme which protects cells against oxidative stress. In recent years, GPX1 gene polymorphisms has been shown to be associated with disease risks and patient survival. Aberrant expression of GPX1 in various cancer types is closely related to oncogenesis or cancer progression. In oral squamous cell carcinoma and renal cell carcinoma, the overexpression of GPX1 is correlated with lymph node metastasis, invasion, and patient survival [10, 11]. In non-small cell lung carcinoma, GPX1 promotes the activation of the PI3K/AKT signaling by regulating ROS levels, leading to chemotherapy drug resistance [12]. Moreover, the expression of GPX1 is elevated in triple-negative breast cancer (TNBC), and stimulates activation of FAK kinase, thereby promoting the adhesion and metastasis of TNBC cells [13]. In hypoxic glioblastoma, GPX1 controls hydrogen peroxide balance and alleviates oxidative stress. Clinical data demonstrates that GPX1 expression is positively correlated with tumor grade and inversely correlated with the overall survival outcome of glioblastoma patients [14]. Hence, the role of GPX1 in oncogenesis or cancer progression is worthy of investigation.

Here, we reported that GPX1 was highly expressed in T315I mutant CML cells compared to BCR/ABL wildtype cells. GPX1 deletion showed reduced-tolerance to nutritional stress by impeding OXPHOS. GPX inhibitor mercaptosuccinic acid (MSA) inhibited CML colony formation and induced cell apoptosis. Hence, GPX1 is a promising therapeutic target for T315I-CML treatment. Future studies should further develop novel strategies on GPX1 for more precision therapy.

## Results

### BCR/ABL-T315I CML cells are tolerant to metabolic stress

**Fig. 1:**
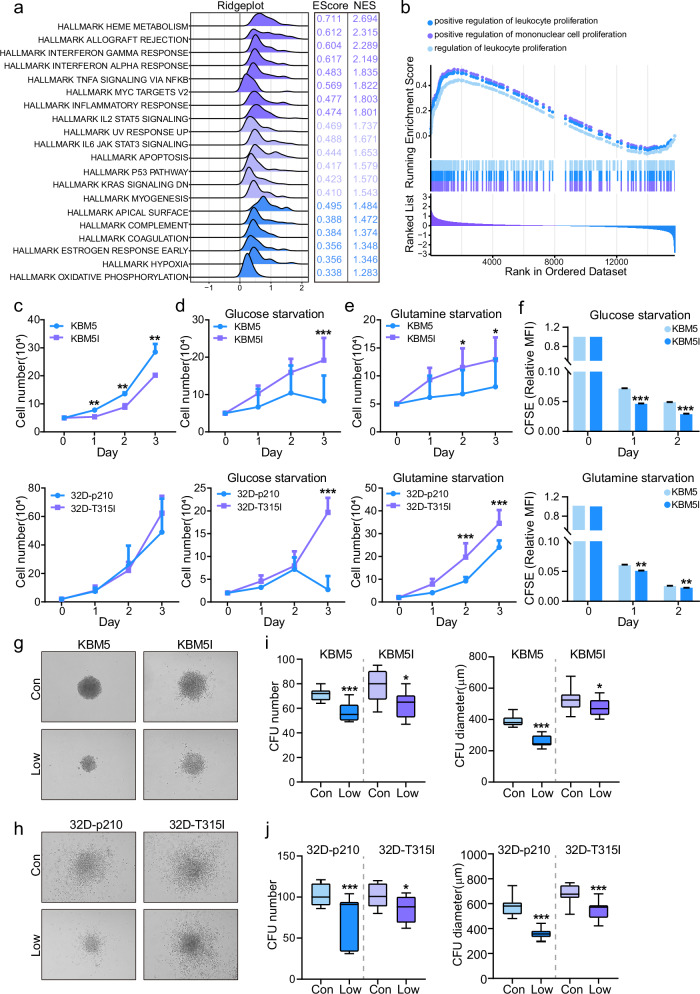
BCR/ABL-T315I CML cells are tolerant to metabolic stress. The bone marrows of 3 BCR/ABL-T315I CML patients and 3 non-T315I patients were collected and subjected to RNA-Seq. GSEA-Hallmark (**a**) and GSEA-KEGG (**b**) analyses were performed. The number of BCR/ABL wildtype and BCR/ABL-T315I cells were counted for three consecutive days under the condition of normal culture system (**c**), glucose deprivation (**d**) or glutamine deprivation (**e**). **f** KBM5 cells and KBM5-T315I cells were stained with CFSE and subjected to flow cytometry. The CFUs of KBM5 and KBM5-T315I (**g**) as well as 32D-p210 and 32D-T315I (**h**) cells under the condition of nutritional deficiency were shown (Con: Control; Low: 1/3 concentration of glucose and glutamine). Colony numbers and diameters between KBM5 and KBM5-T315I cells (**i**), as well as 32D-p210 and 32D-T315I cells (**j**), under the condition of nutritional deficiency, were compared. Data are expressed as mean ± SD. *P < 0.05, **P < 0.01, ***P < 0.001.

To find out the difference of BCR/ABL-T315I induced gene profile, we collected bone marrows from T315I CML patients compared with non-T315I patients, and RNA-Seq analysis was performed. The results showed that BCR/ABL-T315I CML patients displayed hallmark of IL2 STAT5, IL6 JAK STAT3 signaling, P53 pathway and KRAS signaling. Specially, OXPHOS was also enriched, suggesting that T315I altered the metabolism of CML cells towards mitochondrial metabolism (Fig. 1a). Meanwhile, positive regulation of leukocyte proliferation signaling was observed by GSEA (Fig. 1b). We next applied two pairs of cells, KBM5 and KBM5-T315I, as well as 32D-p210 and 32D-p210-T315I, to cell counting. BCR/ABL T315I mutation did not showed short-term proliferative advantage but was associated with change in drug sensitivity (Fig. 1c; Suppl Fig. 1a, b). However, when cells were exposed to the nutritional stress by glucose or glutamine starvation, T315I mutation revealed more resistant to metabolic alternation (Fig. 1d–f; Suppl Fig. 2), implying that KBM5-T315I cells exhibited dominant proliferative capacity against stress. Indeed, after long-term cultivation, the diameter of colony formation units (CFUs) was bigger in T315I cells compared to wildtype cells, although the number of CFUs were comparable (Fig. 1g–j). The above results indicated that BCR/ABL-T315I mutation endowed leukemic cells with the capacity to adapt to metabolic stress.

### BCR/ABL-T315I cells are more likely to depend on mitochondrial metabolism

**Fig. 2:**
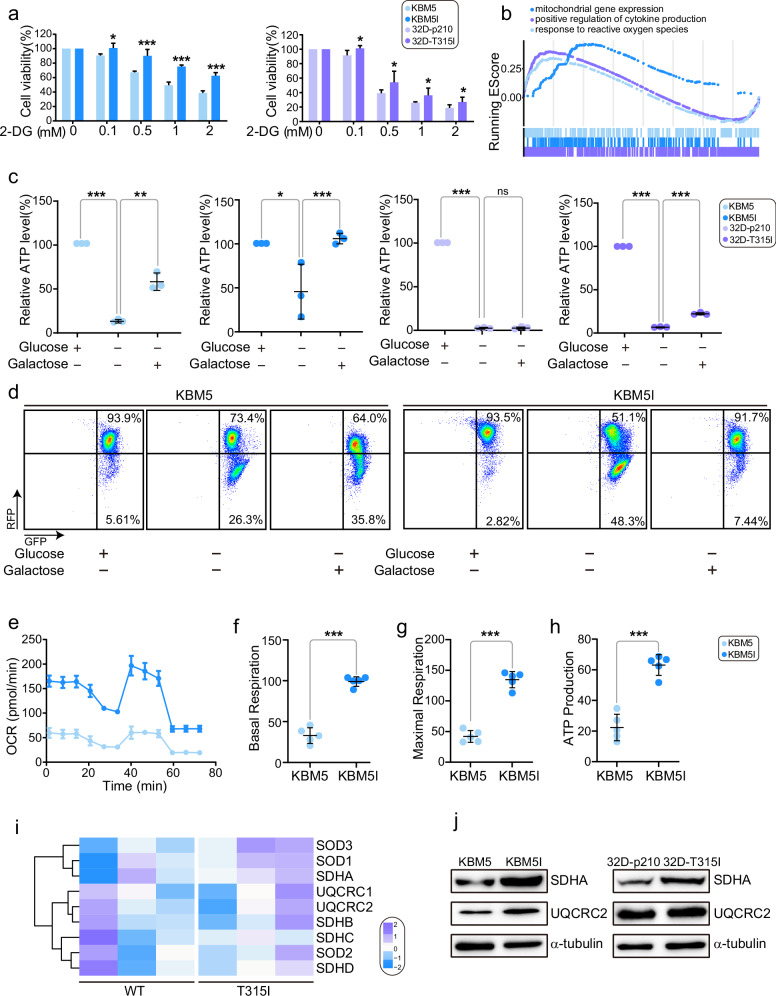
BCR/ABL-T315I CML cells display high OXPHOS. **a** BCR/ABL wildtype and BCR/ABL-T315I cells were treated with 2-DG for 48 h, and CCK-8 assay was performed. **b** RNA-Seq data from BCR/ABL-T315I and non-T315I samples were analyzed by GSEA. ATP level (**c**) and mitochondrial membrane potential (**d**) of BCR/ABL and BCR/ABL-T315I cells cultured under the condition of galactose replenishment were determined. OCR of BCR/ABL and BCR/ABL-T315I cells were measured (**e**) and basal respiration, maximal respiration, as well as ATP production, was shown (**f**–**h**). **i** Heatmap of mitochondrial genes in CML patients from RNA-Seq was displayed. **j** Western blot was used to confirm the expression of mitochondrial proteins. Data are expressed as mean ± SD. *P < 0.05, **P < 0.01, ***P < 0.001.

Since BCR/ABL-T315I mutation conferred resistance to metabolic stress, we then studied whether glycolysis was involved in the process. We tested the effect of 2-deoxyglucose (2-DG), a glycolytic inhibitor that targets hexokinase, on cell viability. As shown in Fig. 2a, 2-DG significantly inhibited the proliferation of KBM5 and KBM5-T315I, as well as 32D-p210 and 32D-T315I cells, in a dose-dependent manner. The different sensitivity of the T315I cells and wildtype cells confirmed that T315I mutation conferred significant tolerance by inhibition of glycolysis and could maintain cell survival through other metabolic pathways. GSEA further showed that mitochondrial gene expression and response to ROS signaling were enriched in T315I patients (Fig. 2b), suggesting that mitochondrial metabolism was altered in T315I cells compared with wildtype cells. Galactose is a tool to mimic glycolytic limitation and forces mitochondrial metabolism [15]. We then observed the recovery of ATP level under the condition of galactose supplement without glucose. The results showed that compared with wildtype cells, T315I cells recovered their ATP level better after the addition of galactose (Fig. 2c). The mitochondrial membrane potential was decreased when the cells were deprived of nutrients such as glucose. Similarly, after galactose replacement, we compared the recovery of mitochondrial membrane potential, and the compensatory increase of mitochondrial membrane potential in T315I mutant cells was also observed (Fig. 2d). Since ATP was mainly produced by OXPHOS in the mitochondria, we used Seahorse XFe96 extracellular flux analyzer to test mitochondrial stress. We found that T315I cells had higher oxygen consumption rate (OCR) than wildtype cells (Fig. 2e), as presented by basal respiration, maximal respiration, as well as ATP production (Fig. 2f–h). Heatmap of CML patients from RNA-Seq showed that some mitochondrial genes were changed between wildtype and T315I, such as succinate dehydrogenase A (SDHA), superoxide dismutase (SOD) and ubiquinol-cytochrome c reductase core protein (UQCRC) (Fig. 2i). Western blot further confirmed that SDHA was highly expressed in T315I cells (Fig. 2j). Above experiments suggested that T315I cells were more likely depend on mitochondrial metabolism, which might lead to increased metabolic tolerance.

### GPX1 is highly expressed in BCR/ABL-T315I cells

**Fig. 3:**
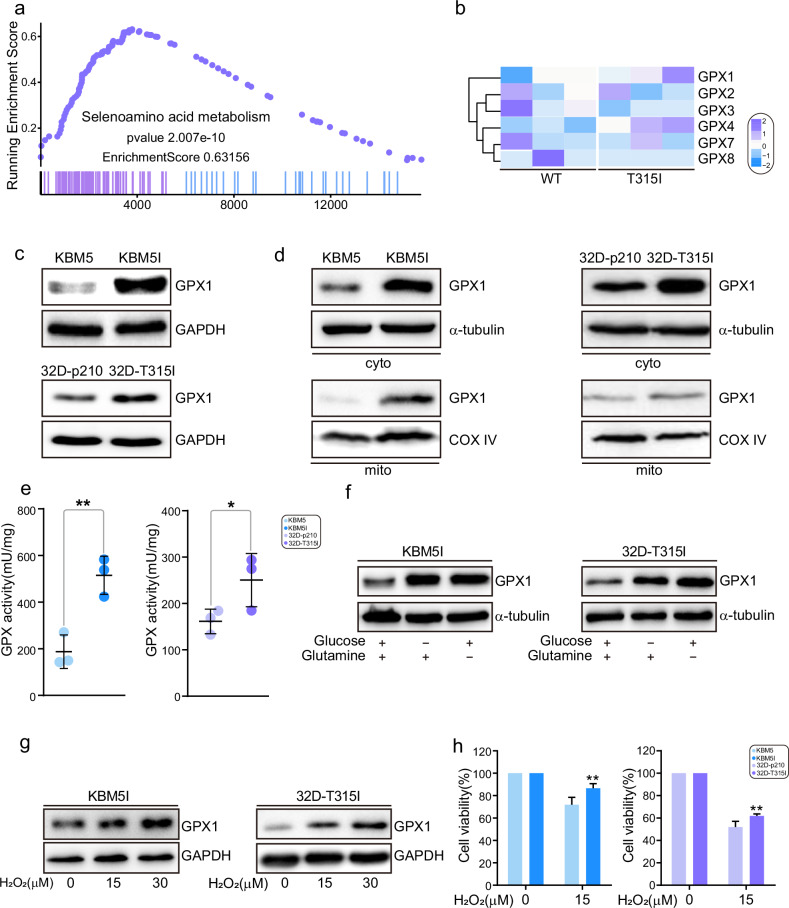
GPX1 is highly expressed in BCR/ABL-T315I cells. GSEA (**a**) and heatmap of GPXs (**b**) from RNA-Seq data of BCR/ABL-T315I CML and non-T315I patients were shown. **c** The expression level of GPX1 was detected by western blot. **d** GPX1 distribution in cytoplasm and mitochondria of BCR/ABL and BCR/ABL1-T315I cells was measured. **e** GPXs enzyme activity of BCR/ABL and BCR/ABL1-T315I cells was determined. **f** KBM5-T315I and 32D-T315I cells were cultured under glucose or glutamine deprivation for 48 h, and the GPX1 protein level was determined by western blot. Cells were treated with different concentrations of hydrogen peroxide for 48 h, and GPX1 expression (**g**) as well as cell viability (**h**) was measured. Data are expressed as mean ± SD. *P < 0.05, **P < 0.01.

Importantly, we also found that selenoamino acid metabolism was increased in T315I patients by GSEA (Fig. 3a). GPX1 belongs GPXs family and is one of the selenocysteine (Sec)-containing proteins [9]. In T315I patients, GPX1 was highly expressed as shown in Fig. 3b. Continently, KBM5-T315I and 32D-T315I cells also displayed high expression of GPX1 (Fig. 3c). Since GPX1 maintains redox balance by controlling ROS level, the localization of GPX1 was detected. Both in KBM5 and 32D cells, GPX1 is located on the cytosol and mitochondria, which showed higher expression in T315I cells (Fig. 3d). The activity of GPXs was consistently increased when BCR/ABL harbored T315I (Fig. 3e). In addition, when T315I cells were exposed to the stress, including glucose or glutamine deprivation, GPX1 was significantly induced (Fig. 3f). H_2_O_2_ stimulation also enhanced GPX1 expression and T315I cells exhibited more tolerance (Fig. 3g, h). These results revealed that GPX1 could be served as a sensor to metabolic stress.

### GPX1 harnesses mitochondrial metabolism in BCR/ABL-T315I cells

**Fig. 4:**
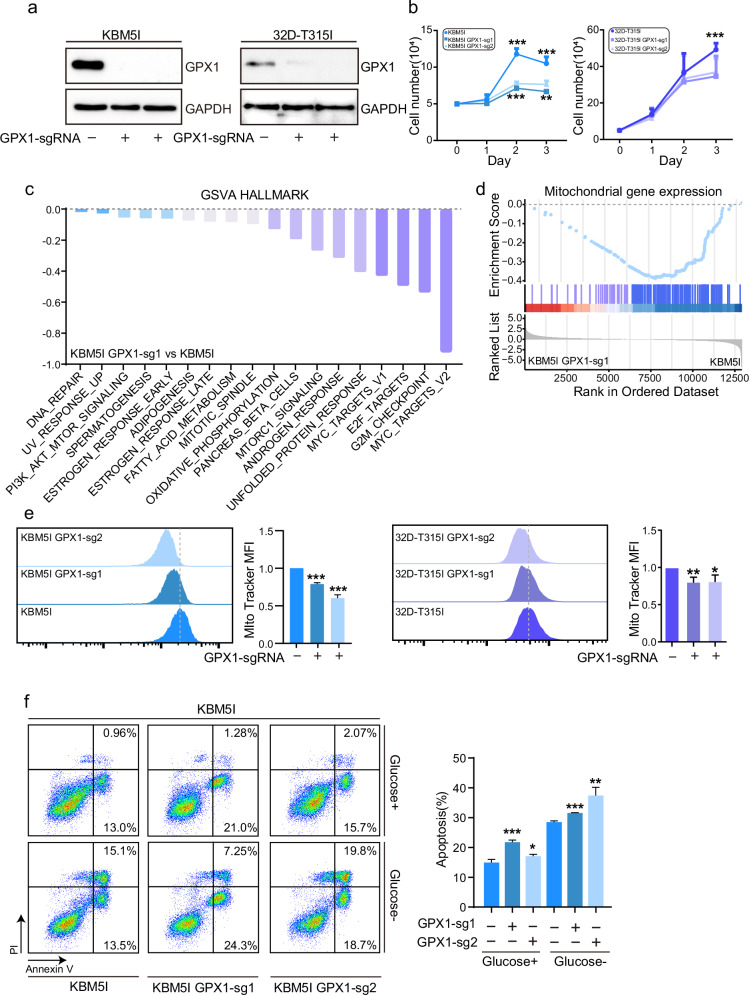
Knockout of GPX1 disturbs mitochondrial metabolism. **a** GPX1-knockout efficiency in KBM5-T315I and 32D-T315I cells was determined by western blot. **b** Control and GPX1-knockout cells were subjected to cell counting under the condition of glucose deprivation. GSVA (**c**) and GSEA (**d**) were performed by RNA-Seq data from control and GPX1-knockout of KBM5-T315I cells. **e** Mitochondrial activity was detected by flow cytometry by MitoTracker Deep Red staining. **f** Apoptosis of control and GPX1-knockout cells under the condition of glucose deprivation was shown. Data are expressed as mean ± SD. *P < 0.05, **P < 0.01, ***P < 0.001.

In order to characterize the function of GPX1 in T315I cells, we knocked out GPX1 by Crispr cas9 (Fig. 4a). As shown in Fig. 4b, GPX1 deletion suppressed the cell viability of both KBM5-T315I and 32D-T315I cells. We then performed RNA-Seq in KBM5-T315I control cells and KBM5-T315I GPX1-knockout cells. GSVA hallmark showed that MYC and E2F signaling, as well as metabolism signaling, were decreased, including OXPHOS (Fig. 4c). GSEA revealed that mitochondrial gene expression was reduced after GPX1 knockout (Fig. 4d), suggesting that GPX1 was essential in mitochondrial OXPHOS. Active mitochondria, as indicated by MitoTracker Deep Red intensity in cells, were reduced in GPX1-knockout cells (Fig. 4e), indicating mitochondria was impaired after GPX1 deletion. Next, we found that GPX1 deletion strongly triggered apoptotic cell death when glucose was deprived (Fig. 4f). Hence, GPX1 could harness cell apoptosis by regulating mitochondrial metabolism.

### Mercaptosuccinic acid overcomes BCR/ABL-T315I-induced drug resistance

**Fig. 5:**
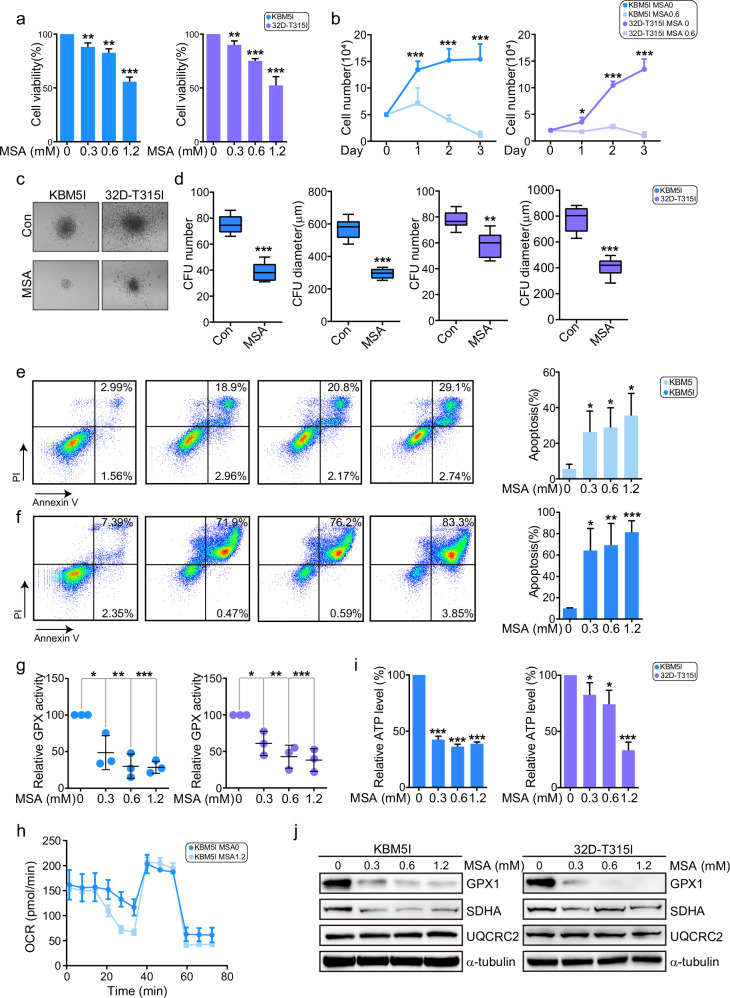
MSA overcomes BCR/ABL-T315I-induced drug resistance. **a** KBM5-T315I and 32D-T315I cells were treated with MSA for 48 h, and the cell viability was determined. **b** T315I cells were treated with MSA for 48 h under glucose deprivation and cell counting was conducted. Colony formation of KBM5-T315I and 32D-T315I cells with 0.075 mM MSA treatment was measured (**c**), and the number and diameter of CFUs were shown (**d**). **e**, **f** KBM5 and KBM5-T315I cells were treated with MSA for 48 h under glucose deprivation, and apoptosis was conducted by flow cytometry. T315I cells were treated with MSA, and GPXs enzyme activity (**g**), OCR (**h**) and ATP level (**i**) were determined. **j** T315I cells were treated with MSA for 48 h and subjected to Western blot. Data are expressed as mean ± SD. *P < 0.05, **P < 0.01, ***P < 0.001.

Moreover, we applied a GPX inhibitor MSA to investigate whether it could restrain BCR/ABL-T315I cells. CCK-8 assay and cell counting assay showed that MSA inhibited cell proliferation in a dose-dependent manner in KBM5-T315I and 32D-T315I cells (Fig. 5a, b). MSA also suppressed CFU number and diameter in T315I cells (Fig. 5c, d). Furtherly, apoptosis was induced by MSA in KBM5 and KBM5-T315I cells under glucose deprivation, especially in the mutant cells (Fig. 5e, f). Meanwhile, MSA significantly inhibited GPXs activity (Fig. 5g). Mechanistically, OCR and ATP level were downregulated (Fig. 5h, i), and SDHA was decreased (Fig. 5j). Thus, MSA could eliminate T315I cells and overcome imatinib resistance through breaking mitochondrial balance. Metformin, a hypoglycemic agent, has shown anti-cancer activity in a variety of tumor types. It has been reported to inhibit the function of mitochondrial electron transport chain complex I, reduce oxygen consumption and mitochondrial ATP synthesis [16]. In our study, BCR/ABL-T315I mutant cells were sensitive to metformin (Suppl. Fig. 3a). Moreover, metformin could increase cell sensitivity to imatinib (Suppl. Fig. 3b). Therefore, impeding mitochondrial homeostasis could overcome imatinib resistance caused by the T315I mutation.

## Disscussion

In most CML patients with BCR/ABL, the disease can be kept under control with imatinib or with second or third-generation TKI. Nevertheless, a portion of patients fail to respond to all available TKIs and experience relapse with poor outcomes. Kinase domain mutation of the BCR/ABL fusion gene, especially the T315I mutation, still pose a bottleneck in CML treatment. During the past few years, tremendous attempts have been made to search for novel promising therapeutic targets and new drugs on CML mutant cells to overcome TKI resistance. Alternative pathways are being explored for the treatment of CML, as BCR/ABL is not the only signaling pathway governing the growth and proliferation of CML cells. Blocking the hedgehog pathway impairs hematopoietic stem cell renewal and decreases BCR/ABL-induced CML and drug resistance [17]. Targeting the NF-κB and β-catenin pathways may offer opportunities to overcome imatinib resistance [18]. Our previous study also shows that inhibition of AURKA could overcome drug resistance caused by the T315I mutation [19]. We here showed that GPX1 was highly expressed in T315I CML cells, and mediated cellular metabolism and growth. Moreover, we provided evidence that the MSA could target GPXs and eliminate T315I mutant cells.

Alteration in energy metabolism is an important hallmark of leukemia cells while gene mutation plays a crucial role in the reprogramming of tumor metabolism. For instance, the internal tandem duplication (ITD) mutation in the Fms-like tyrosine kinase 3 gene (FLT3/ITD) induces metabolic alteration and makes leukemia cells highly dependent on glycolysis [20]. Similarly, mutation in isocitrate dehydrogenase-1 (IDH1) alters the enzymatic function and results in the production of oncometabolite 2-hydroxyglutarate (R-2-HG), leading to leukemogenesis and hematopoietic differentiation block [21]. Kras mutation (G12D) promotes pancreatic ductal adenocarcinoma (PDAC) through regulation of anabolic glucose metabolism. G12D triggers glucose uptake and redirects glucose intermediates towards the hexosamine biosynthesis and pentose phosphate pathways [22]. Imatinib treatment of BCR/ABL-driven leukemic cells results in alternation of glycolytic enzymes and reactivated mitochondrial OXPHOS [23]. Indeed, tyrosine kinase activation driven by BCR/ABL causes metabolic modulation and leads to a significant upregulation of ROS, which is likely due to mitochondrial dysfunction [24]. BCR/ABL-T315I mutation leads to the downregulation of genes related to glycolysis pathways and a reduction in ROS levels [25]. We found here that the metabolism of BCR/ABL-T315I CML patients was altered towards OXPHOS. Besides enhancing the long-term proliferation, T315I mutation conferred CML cells the tolerance to metabolic stress, such as glucose or glutamine derivation.

GPX represents one of the crucial antioxidant enzyme families in protecting cells from lethal oxidative stress and maintaining redox balance [26]. In recent years, GPX1 has been well identified as closely linked to the pathogenicity of various cancer types. GPX1 is overexpressed in thyroid cancer, glioma and skin melanoma, as well as leukemia [9]. It is also highly expressed in renal cell carcinoma (RCC) and shows promise as a biomarker for RCC diagnosis and prognosis [10]. In TNBC cells, GPX1 depletion prevents cell migration and reduces metastasis by reducing FAK/c-Src activation [13]. GPX1 can also mediate redox homeostasis and tumor progression, which are regulated by glutamate dehydrogenase 1 (GDH1) [27]. In leukemia, high GPX1 expression is associated with a poor prognosis of AML patients [28]. Previous report has indicated that GPX1 interacts with c-ABL and functions as a substrate for c-ABL-mediated phosphorylation on Tyr-96, thereby protecting cells from oxidative stress [29]. GPX1 expression can be induced by imatinib and appears to be dependent on BCR/ABL expression [30]. Therefore, the expression of GPX1 may have a profound effect on the clinical outcome in BCR/ABL-induced CML. Surprisingly, we have found that T315I mutant cells have a higher tolerance to nutritional stress with high expression of GPX1. Cancer cells commonly possess upregulated intracellular antioxidant system due to increased level of cell metabolism, accommodating to oxidative stress [31]. Different cell types may respond differently to nutritional stress. For example, glucose deprivation causes GPX1 degradation and activates ROS-dependent autophagy in PDAC [32]. Conversely, we found that glucose or glutamine deprivation, as well as H_2_O_2_ treatment, could induce GPX1 expression, indicating that GPX1 could respond to environmental changes in CML cells. We then knocked out of GPX1 in the T315I cells and found OXPHOS signaling were downregulated in T315I mutant cells. Mitochondrial metabolism was disturbed as assessed by mitochondrial activity. Additionally, after GPX1 deletion, cells were more sensitive to nutritional deprivation, indicating that GPX1 was essential for cells to endure nutritional stress.

As targeting OXPHOS in several cancer types, including leukemia, has become apparently important, the development of clinically applicable OXPHOS inhibitors has aroused interest. IDH inhibitors [33] and BCL-2 inhibitor venetoclax [34] have shown effective benefit by influencing cellular metabolism. GPX1 is overexpressed in various tumors to induce drug resistance, and many studies have been exploring inhibitors for cancer therapy. Pentathiepins induce a loss of mitochondrial membrane potential and oxidative stress in cancer cells, leading to DNA strand breaks and apoptosis [35]. MSA competes with glutathione for binding to the Sec active site of GPX [36]. In this study, MSA prohibited CML cell proliferation and increased cell apoptosis under glucose starvation. MSA also significantly reduced cellular colony formation. Mechanistically, GPX1 was of vital importance for decreasing OCR, and reducing the expression of mitochondrial proteins, which were essential for mitochondrial redox homeostasis. Therefore, GPX1 presented itself as a promising therapeutic target in CML treatment, particularly for those with the T315I mutation. Previous study shows that the metabolic vulnerability of imatinib-resistant cells can be targeted by a combination of 2-DG and imatinib as an alternative treatment through induction of autophagy [37]. Another study reveals that the resistance of T315I mutation is overcome by mitochondrial damage-induced apoptosis through targeting VDACs [38]. We showed that metformin, as an inhibitor of mitochondrial complex I, could induce synergistic effect with imatinib to suppress cell proliferation, providing evidence that ‌impediment of mitochondrial metabolism could overcome imatinib resistance in CML.

In the present study, we revealed that T315I mutant cells were tolerant to metabolic stress. GPX1 was highly expressed in T315I mutant cells to enhance mitochondrial metabolism and respond to nutritional stress. MSA was responsible for the disturbance of mitochondrial redox homeostasis and reduction of the colony formation ability in T315I mutant cells. Therefore, GPX1 was explicit to be a potential therapeutic target for overcoming drug resistance induced by T315I mutation.

## Materials and methods

### Reagents

MSA and metformin were purchased from Sigma (St. Louis, MO, USA) and dissolved in sterilized deionized water at a concentration of 1 M to prepare a stock solution. 2-deoxy-D-glucose, oligomycin (Merck, Darmstadt, Germany), Carbonyl cyanide 4-(trifluoromethoxy) phenylhydrazone (FCCP) and Rotenone (Topscience, Shanghai, China), were dissolved in DMSO to prepare stock solutions, respectively. Antimycin (Biovision, Milpitas, CA, USA) was dissolved in ethanol with a concentration of 20 mM. RPMI-1640 culture medium without glucose and glutamine was purchased from Procell Company (Wuhan, China).

### Cell culture and plasmid transfection

KBM5 and KBM5-T315I (KBM5I) cell lines were provided by Professor Peng Huang (Sun Yat-sen University Cancer Center). 32D cells were purchased from the American Type Culture Collection. Cells were cultured in RPMI 1640 (Thermo Fisher Scientific, Massachusetts, USA) with 10% FBS in a 37 °C humidified incubator with 5% CO_2_. The 32D cells were infected with a retrovirus expressing pMSCV-IRES-BCR/ABL (p210) and the T315I mutant BCR/ABL (p210-T315I). LentiCRISPR v2 containing sgRNA targeting GPX1 was used to construct GPX1-knockout cells. The sgRNA sequences were shown: GPX1-sgRNA-1 (human): GGCGTCCCTCTGAGGCACCA; GPX1-sgRNA-2 (human): CGAGAAGGCATACACCGACT; GPX1-sgRNA-1 (mouse): CTCGGTGTAGTCCCGGATCG; GPX1-sgRNA-2 (mouse): GCTCGAACCCGCCACCAGGT.

### Cell proliferation and viability assay

The trypan blue exclusion method was used to count live cells under different culture condition or drug treatment. Cell viability was conducted by using the CCK-8 reagent (ApexBio Technology, Houston, TX, USA). Specifically, the cells were cultured in a 96-well plate with 10,000 cells per well. After incubation for 48 h with different treatment in a 37 °C incubator, the absorbance at 450 nm was determined after adding 10 μL of CCK-8 reagent for 4 h.

Cell proliferation was detected using CFSE (Beyotime, Shanghai, China). Appropriate amount of cells were resuspended using serum-free RPMI 1640 culture medium with 0.5 μM CFSE, and incubated at 37 °C for 30 min. Then the supernatant was removed and cells were resuspended in RPMI 1640 medium with 10% FBS and left on ice for 5 min. After that, cells were washed and cultured for 2 days and a flow cytometer was applied to detect the CFSE fluorescence intensity.

### Colony formation assay

Briefly, cells were treated with different condition, and cultured in a final concentration of 0.9% methylcellulose. After 10 days, the colonies were counted, and then photographed by an inverted microscope. Image-Pro Plus software was applied to measure colony diameters.

### Apoptosis assay

Cells were collected and washed with PBS after different treatment for indicated time. Subsequently, cells were resuspended in 100 μL of 1×binding buffer. Annexin V and PI staining solution (Vazyme, Nanjing, China) were added separately and incubated for 15 min at room temperature in the dark. Next, 400 μL of 1× binding buffer was added to resuspend cells, and flow cytometry was performed.

### Western blot analysis

Cells were collected and lysed with RIPA lysis buffer, supplemented with protease and phosphatase inhibitors. The cell lysate was then centrifuged, separated by SDS-PAGE and transferred onto nitrocellulose membranes. The membrane was subsequently blocked with TBST containing 5% BSA for 1 h at room temperature, and incubated with the corresponding primary antibodies overnight at 4 °C, including GPX1 (ab22604) (Abcam, Cambridge, MA, USA); COX IV (4850), SDHA (11998) and α-tubulin (2144) (Cell Signaling Technologies, Danvers, MA, USA); and UQCRC2 (sc-390378) and GAPDH (sc-25778) (Santa Cruz, Dallas, TX, USA). Following this, the membrane was incubated with a peroxidase-conjugated secondary antibodies for 1 h. The protein was detected using an enhanced chemiluminescence reagent (Vazyme, Nanjing, China).

### Measurement of ATP production

ATP level was measured following the instruction provided by the ATP bioluminescence assay kit (Biovision, Milpitas, CA, USA). Briefly, cells were cultured in a 6-well plate at a density of 2.5 × 10^5^ cells/mL. After 48 h, cells were collected, washed twice with PBS, and lysed using the reaction buffer. The lysate was then subjected to centrifugation, and the supernatant was used for ATP level determination by fluorescence measurement. ATP level was calculated based on a standard curve and normalized to cell numbers or protein concentration.

### Detection of mitochondrial membrane potential

Mitochondrial membrane potential was determined by JC-1 staining (MedChemExpress, Monmouth Junction, NJ, USA). Briefly, cells were collected, washed with serum-free RPMI 1640 medium, and then stained with JC-1 for 30 min at 37 °C. Mitochondrial membrane potential was measured by flow cytometry.

### Measurement of mitochondrial activity

MitoTracker Deep Red (Thermo Fisher Scientific, Massachusetts, USA) was employed for measuring mitochondrial activity. Briefly, cells were collected and washed twice with PBS before being resuspended in serum-free RPMI 1640 medium. Subsequently, the cells were incubated with MitoTracker Deep Red at a final concentration of 0.5 μM for 30 min in a dark 37 °C incubator. Following this, the staining was stopped using serum-free RPMI 1640 medium, and the cells were washed twice and resuspended for flow cytometry analysis.

### GPX enzyme activity assay

GPX enzyme activity was assessed following the instruction provided by the GPX enzyme activity assay kit (Beyotime Biotechnology, Shanghai, China). Firstly, 2 × 10^6^ cells were collected, washed twice with PBS, and then resuspended in homogenization buffer containing 1% PMSF. Following this, the cell lysate was homogenized and subsequently centrifuged at 12,000 rpm for 10 min at 4 °C. The supernatant was used to measure the absorbance at 340 nm. Ultimately, the enzyme activity was determined based on the standard curve and then normalized based on the protein concentration.

### Exploration of oxygen consumption rate

CML cells were cultured in a 96-well culture microplate pre-coated with Cell-Tak Cell and Tissue Adhesive (Corning Incorporated, Corning, NY, USA). The plate was then incubated at 37 °C for 3 h. Following the incubation, the culture medium was removed, and the cells were washed. Subsequently, assay medium was added into 96-well plate. The OCR was measured by Seahorse XFe96 analyzer according to the manufacturer’s instruction and then normalized based on the cell numbers.

### RNA sequence analysis

Bone marrow mononuclear cells (BMMCs) were collected from CML patients at the Department of Hematology, Nanfang Hospital. Total RNA of cells was extracted using the TRIzol reagent (Thermo Fisher Scientific, Massachusetts, USA), and RNA-Sequence (RNA-Seq) was performed by Novogene (Beijing, China). RNA-Seq of GPX1-knockout KBM5-T315I cells or control cells was conducted by IGE Biotechnology (Guangzhou, China). Differential genes and enriched pathways were analyzed using edgeR and Limma in the R platform.

### Statistical analysis

The data were analyzed and graphed using GraphPad Prism 8 software and the results were presented as mean (±SD). An unpaired t-test for comparison of two groups was performed for data analysis.

## Supplementary information

suppl. Figure 1

suppl. Figure 2

suppl. Figure 3

suppl. Material 4

Western blot raw data

## Data Availability

The datasets used and/or analyzed during the current study are available from the corresponding author upon reasonable request.
