# Supplementary figures and images for: GPX1 confers resistance to metabolic stress in BCR/ABL-T315I mutant chronic myeloid leukemia cells
Source: Cell Death Discov. 2025 May 9;11:229. doi: 10.1038/s41420-025-02502-z (PMC12064725; doi:10.1038/s41420-025-02502-z)

b

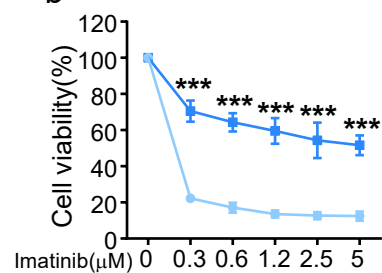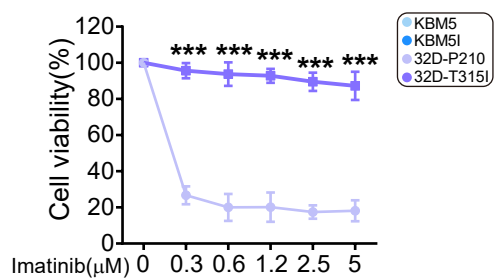

Supplement: Supplementary file 1 — suppl. Figure 1 [file 41420_2025_2502_MOESM1_ESM.pdf]

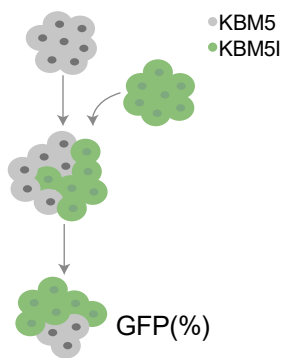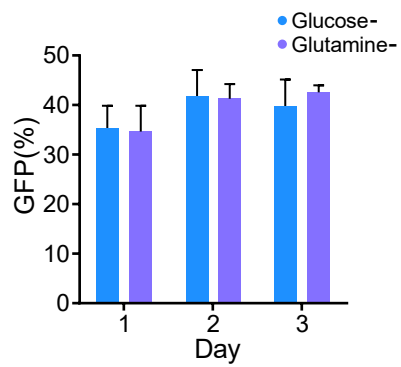

Supplement: Supplementary file 2 — suppl. Figure 2 [file 41420_2025_2502_MOESM2_ESM.pdf]

a

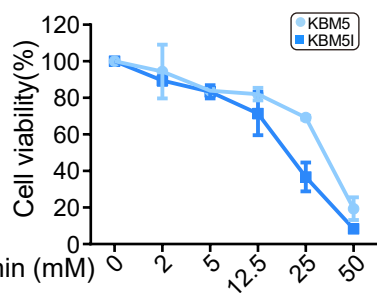

b

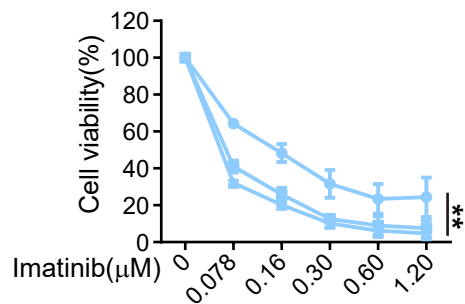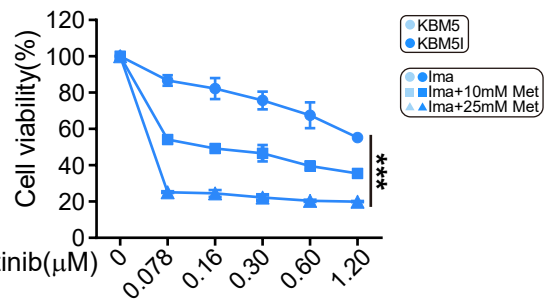

Supplement: Supplementary file 3 — suppl. Figure 3 [file 41420_2025_2502_MOESM3_ESM.pdf]

**Fig.2j**

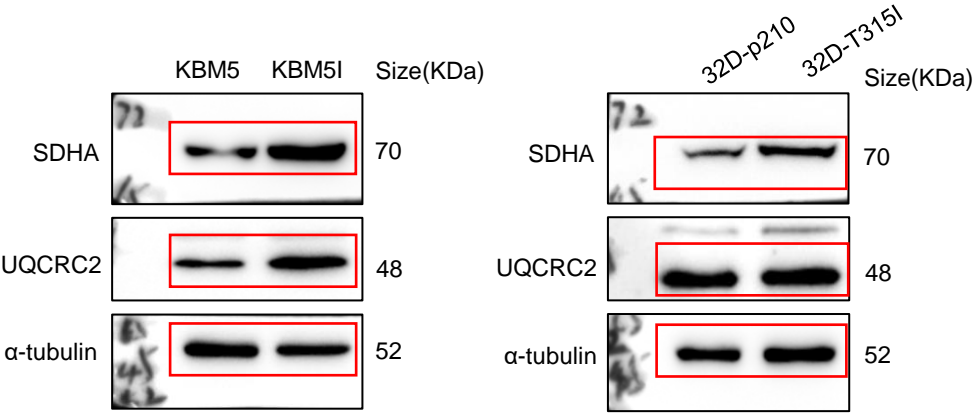

**Fig.3c**

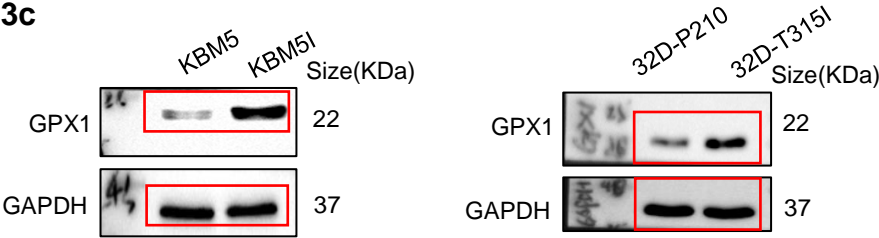

**Fig.3d**

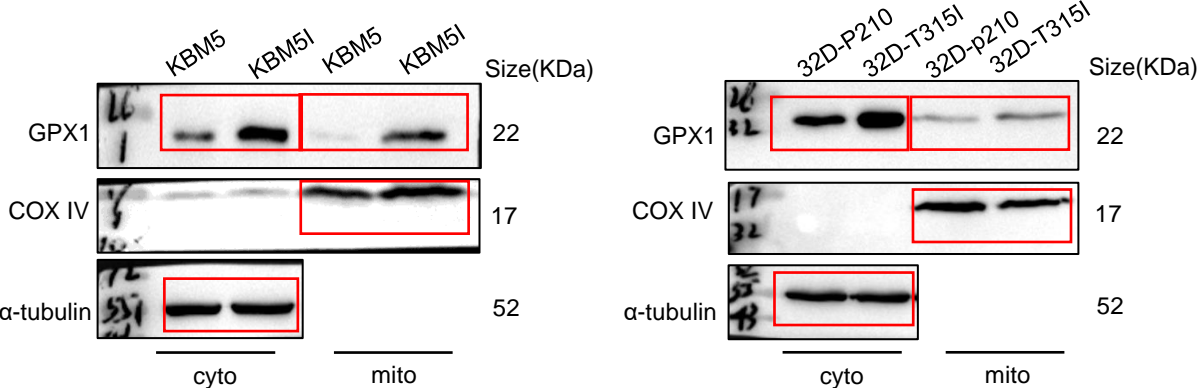

**Fig.3f**

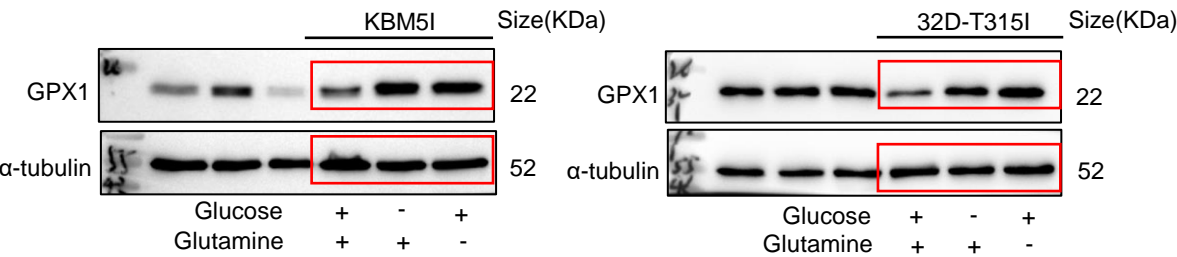

**Fig.3g**

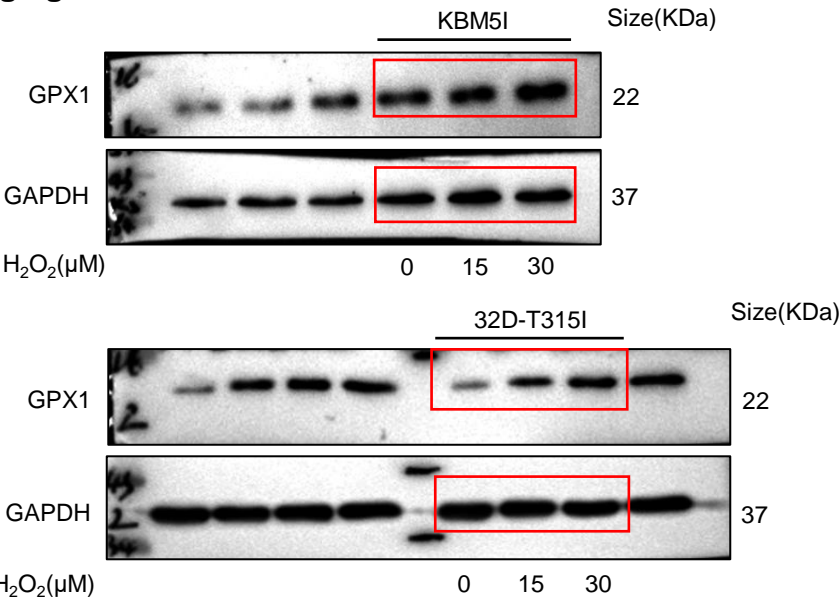

**Fig.4a**

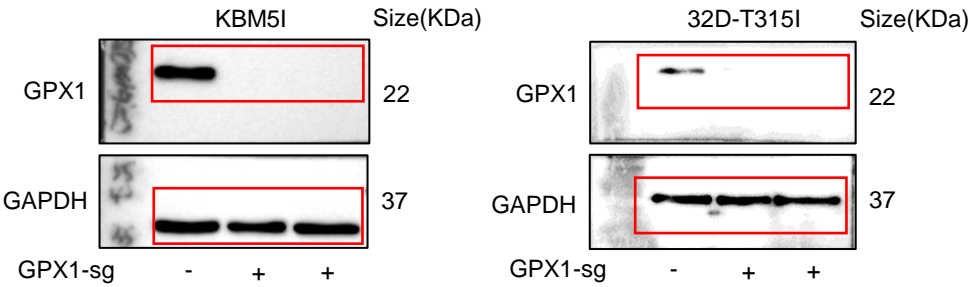

**Fig.5j**

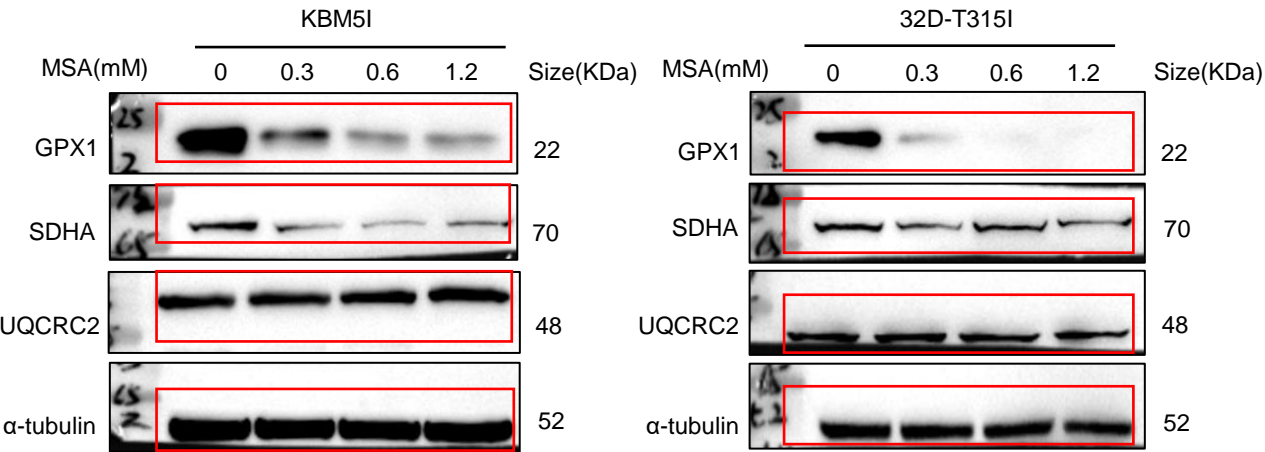

Supplement: Supplementary file 5 — Western blot raw data [file 41420_2025_2502_MOESM5_ESM.pdf]
