# Supplementary material for: GPX1 confers resistance to metabolic stress in BCR/ABL-T315I mutant chronic myeloid leukemia cells
Source: Cell Death Discov. 2025 May 9;11:229. doi: 10.1038/s41420-025-02502-z (PMC12064725; doi:10.1038/s41420-025-02502-z)
Supplement: Supplementary file 4 — suppl. Material 4 [file 41420_2025_2502_MOESM4_ESM.docx]

**Supplemental Material Figure Legends**

**Suppl Figure 1 BCR/ABL-T315I cells are resistant to imatinib.** (a) BCR/ABL wildtype and BCR/ABL-T315I cells were subjected to PCR for BCR/ABL expression. (b) Cells treated with imatinib for 48 h were subjected to CCK-8 assay. Data are expressed as mean±SD. ****P*<0.001.

**Suppl Figure 2 BCR/ABL-T315I cells are resistant to metabolic stress.** KBM5-T315I cells expressing green fluorescent protein (GFP) were established. Then, KBM5 and KBM5I-GFP cells were mixed at a ratio of 1:1 and cocultured for 3 days. The proportion of GFP-positive cells was assessed using flow cytometry.

**Suppl Figure 3** **Metformin increases cell sensitivity to imatinib.** (a) The comparison of sensitivity to metformin between KBM5 and KBM5-T315I cells by CCK-8 assay was shown. (b) The inhibitory effect of metformin combined with imatinib on KBM5 and KBM5-T315I cells was detected by CCK-8 assay. Data are expressed as mean±SD. ***P*<0.01, ****P*<0.001.
